# Editorial focus: understanding off-target effects as the key to successful RNAi therapy

**DOI:** 10.1186/s11658-019-0196-3

**Published:** 2019-12-09

**Authors:** Rafal Bartoszewski, Aleksander F. Sikorski

**Affiliations:** 1 Department of Biology and Pharmaceutical Botany, Medical University of Gdansk, Gdansk, Poland; 2 Department of Cytobiochemistry, Faculty of Biotechnology, University of Wroclaw, Wroclaw, Poland

**Keywords:** ncRNAs, microRNAs, siRNAs, RNAi therapy, RNAi drug candidates, Off-target effects

## Abstract

With the first RNA interference (RNAi) drug (ONPATTRO (patisiran)) on the market, we witness the RNAi therapy field reaching a critical turning point, when further improvements in drug candidate design and delivery pipelines should enable fast delivery of novel life changing treatments to patients. Nevertheless, ignoring parallel development of RNAi dedicated in vitro pharmacological profiling aiming to identify undesirable off-target activity may slow down or halt progress in the RNAi field. Since academic research is currently fueling the RNAi development pipeline with new therapeutic options, the objective of this article is to briefly summarize the basics of RNAi therapy, as well as to discuss how to translate basic research into better understanding of related drug candidate safety profiles early in the process.

## Introduction

Francis Crick’s 1957 central dogma lecture changed the course of modern biology and the pharmaceutical industry by placing proteins at the end of the biological information transfer [1–3]. Consequently, perturbations in protein levels and function contribute to pathomechanisms of human diseases, despite their molecular, genetic and physiological origins. Hence, restoring human protein homeostasis has become one of the main goals of research into post-genomic therapeutic strategies. However, it quickly became clear that only some disease-related proteins have the ability to bind small chemical molecules, being potential drugs. Indeed, as estimated in the early 2000 s, among the approximately 3000 disease-related proteins encoded in the human genome, only 600-1500 are potential small-molecule drug targets (proteins with enzymatic function or a conformation that is accessible to traditional drug molecules) [4–6]. Similarly, the highly specific, protein-based drugs including monoclonal antibodies are mainly limited to cell-surface receptors or circulating proteins [7, 8]. Notably, about 80% of the proteins involved in human diseases execute two or more biochemical functions [9], and thus their precise chemical targeting can be very difficult or impossible due to potential adverse effects. Furthermore, pharmacologically relevant small molecule-mediated therapeutic effects often rely on maximizing drug-receptor effects (at above 90% target engagement), requiring high dosing levels and thus reduced safety [10]. Thus, the discovery and development of alternate therapeutic strategies addressing and exploiting chemically “undrugabble” proteins have remained a challenge for the industry.

The 2006 Nobel prize crowned the discovery of RNA interference (RNAi) [11] as a pathway in which small non-coding RNA molecules, by controlling mRNA stability and translation, modulate protein cellular levels. Furthermore, subsequent reports that short (21 and 22 nucleotide) double stranded RNAs (dsRNAs) may enter the RNAi silencing pathway in mammalian cells [12–14] opened new prospects for the pharmaceutical industry. Initially, the opportunity for rational drug design to treat diseases that were once thought to be untreatable was well received by drug developers. However, subsequent unsuccessful clinical trials revealed numerous limitations of RNAi application, including: dose-limiting and immune-related toxicities, insufficient therapeutic efficacy, poor metabolic stability, as well as off-targets effects [15–20]. Hence, despite confirming efficient RNAi therapy in humans, the mainstream pharmacological industry withdrew from the RNAi field in the 2010s [20–22].

However, despite this excessive skepticism toward RNAi therapy, in August 2018 a small interfering RNA (siRNA) against transthyretin (*TTR*) mRNA, ONPATTRO (patisiran) was proven to be an effective therapy for hereditary transthyretin amyloidosis (hATTR) and approved as the first RNAi drug by both the US Food and Drug Administration (FDA) and the European Medicine Agency (EMA) [23–25]. Furthermore, multiple RNAi drug candidates are currently progressing through clinical trials, with many of them excelling and reaching phase III [25]. Hence, we witness the RNAi therapy field reaching a critical turning point, when further improvements in drug candidate design and delivery pipelines should enable fast delivery of novel life changing treatments to patients. Furthermore, microRNA (miRNA) based drug candidates promise not only elimination of erratic proteins (such as siRNA), but also provide tools to restore missing proteins to physiological levels [26–44]. Importantly, since mammalian miRNAs are not perfectly complementary to their target mRNA sequences and have multiple targets, this directly translates into a higher attrition rate in related drug discovery. Hence, ignoring parallel development of RNAi dedicated in vitro pharmacological profiling [45] aiming to identify undesirable off-target activity may slow down or even halt progress in the RNAi field.

Since academic research is currently fueling the RNAi development pipeline with new therapeutic options, the objective of this article is to briefly summarize the basics of RNAi therapy, as well as to discuss how to translate basic research into better understanding of related drug candidate safety profiles early in the process.

### RNA interference

RNA interference is a native gene silencing pathway of most eukaryotic cells that utilizes non-coding RNA (ncRNA) molecules (produced by various mechanisms) to obtain efficient post-transcriptional repression of homologous sequences [46–48]. ncRNA molecules act on specific mRNAs through short guide strands that recognize complementary bases in the target RNAs. With an 8 nucleotide (nt) long region called the “seed sequence,” the guide strands must have significant homology to their target strand(s) in order to allow the RNAi mechanism to affect gene expression. The guide strands, depending on their biogenesis and actions on the intended mRNAs, can be broken up into the three categories of RNAi.

(i) miRNAs are short (approx. 22 nt) endogenous non-coding single substrates for the RNAi machinery [49]. microRNAs are encoded in both introns and intergenic clusters and these genes are first transcribed by RNA polymerase II into long primary miRNA (pri-miRNA) transcripts. Next, the pri-miRNA are processed by the the double-strand-specific ribonuclease Drosha-DGCR8 complex transcripts into precursor miRNA (pre-miRNA) stem loop structures [50] that, following their transport to the cytoplasm, are further dissected by the Dicer RNAase III endonuclease to deliver mature 21-23 nucleotide microRNAs [50–56]. Notably, an alternate, Dicer-independent miRNA biogenesis pathway has also been reported [57]. Mature miRNAs strands are associated with Argonaute 2 (Ago2) containing RNA-induced silencing complexes (RISC) that can diminish a specific target mRNA by Ago2-catalyzed degradation of the mRNA and down regulate specific target gene expression via either reducing the transcript levels or by translational repression [52–56, 58–63]. Notably, in humans, only Ago2 carries catalytic cleavage activity [64, 65]. microRNAs perceive their target mRNAs through base-pairing interactions between nucleotide numbers 2 and 8 of the miRNA (the seed sequence) and the complementary nucleotides in the 3′-untranslated region (3′-UTR) of the mRNAs [66–69]. Importantly, nuclear mammalian miRNAs mediated nuclear chromatin silencing at specific loci by base pairing to nascent transcripts has also been reported [70–72].
(ii) Small interfering RNAs (siRNA) being ∼21-22 bp long dsRNA with 3′ two-nucleotide overhangs originate from cytosolic Dicer mediated processing of 30 to 100 bp dsRNA that are either transcribed from cellular genes or introduced into the cells by infecting pathogens, or artificially via transfection or transduction by a viral-derived vector [12, 47, 73, 74]. siRNA interacts with and activates RISC (Ago2 cleaves and releases the “passenger” siRNA strand (sense strand), while the “guide” strand (antisense strand) remains associated with the complex) [73, 74]. The single “guide” strand of siRNA directs the specificity of the mRNA target recognition and cleavage by Ago2 by intermolecular base pairing [74]. mRNA targets that bind the “guide” strand with perfect or near-perfect complementarity are then degraded by Ago2, and thus specific gene expression silencing is obtained [27, 75]. In some cases, however, imperfect complementarity between the “guide” strand and target mRNA may mimic miRNAs’ mediated translational repression [76]. Importantly, RISC can also mediate transcriptional gene silencing using the siRNA specificity to direct silent chromatin modifications over homologous DNA loci [77]. Natural siRNAs likely originated as a defense mechanism against viruses and foreign DNA elements, allowing their elimination [47, 78].
(iii) piwi-interacting RNAs (piRNAs) are small, ∼23-30-nucleotide, endogenous RNAs that are issued from long single stranded precursors-a Dicer-independent mechanism [79]-and serve as repressors of transposable elements (TE) [79]. Hence, piRNA safeguard mammalian germ cells from deleterious effects of transposons and preserve chromatin structure [79]. piRNAs guided silencing is analogous to the other RNAi mechanism in that piRNAs guide PIWI proteins to target mRNAs through RNA base pairing and the mRNAs are then dissected by the endonuclease activity of the PIWI proteins [80–83]. Although initial studies assigned piRNAs activity with mammalian germ cells, emerging evidence suggests that they may be functional in somatic cells as well [79, 81, 82, 84–91]. However, despite the increasing interest in piRNA mediated mechanisms, knowledge about their functional roles remains fairly limited. Furthermore, related experimental approaches are limited by the plethora of unique piRNAs sequences [92–97]. and the lack of easily available verified piRNAs analogs and inhibitors. Hence, although piRNAs may eventually be included in RNAi therapy, this is not going to be quick or easy process.

siRNAs are highly specific with only one mRNA target and generally allow effective gene silencing. This makes siRNAs the leading branch of the developing specific RNAi therapies. However, these therapies are limited to the elimination of target proteins. In contrast, miRNAs have multiple targets, and consequently specific miRNAs can modulate transcriptional networks involving diverse autonomous targets such as transcription factors [98–100], and thus avoiding off-target effects can be extremely difficult. Furthermore, despite the fact that some miRNAs have large switch-like effects reported under conditions of stress or disease [101–113], these RNAs instead modulate protein levels than serve as strong post-transcriptional repressors [114]. Hence, the miRNA-based drug discovery process seems very challenging, as is reflected by the limited number of drug candidates undergoing clinical trials [20, 25–27]. Nevertheless, miRNA and their analogs (antagomiRs) and agonists for RNA (target protectors/block-miRs) provide a therapeutic opportunity for not only eliminating proteins but also restoring their physiological levels and thus should be considered as the future of RNAi therapies [115].

### Design of RNAi drug candidates

Although the therapeutic potential of RNAi drugs is evident, their formulation must overcome different sets of hurdles impeding their development into clinical use, including: off-target activity, immunogenic reactions to foreign dsRNA, immunogenic as well as non-immunogenic effects of delivery chemicals, specific tissue delivery, as well as obtaining desired drug candidate pharmacokinetics and bioavailability (stability, competition with endogenous RNA, cellular uptake, endosomal escape) [19, 25–27, 35, 41]. To date, numerous design and delivery strategies have been developed to address these obstacles and to enhance RNAi drug candidate efficacy and specificity.

### Entering the RNAi pathway

RNAi drug candidates triggered by synthetic RNAs channel into the RISC pathway at the cytoplasmic stage. Minimal 15-30 bp, fully complementary dsRNAs or short hairpin RNAs (shRNAs) are most commonly used. dsRNAs longer than 30 bp have increased propensity for off-targeting and inducing nonspecific cytotoxicity via activating interferon pathway [116]. dsRNAs shorter than 15 bp are not recognized by RNAi machinery. Importantly, dsRNAs shorter than 21 bp do not require Dicer processing prior to association with RISC [117, 118]. However, it has been reported that Dicer processing of RNAi drug candidates results in their increased potency and better specificity (it has been reported that dsRNAs with 27 nucleotides are up to 100 times more efficient than typical siRNAs with 21 nucleotides) [119–122]. In contrast, dsRNAs that bypass Dicer processing provide the opportunity for more extensive chemical modification of such RNAs and thus obtaining better metabolic stability [123]. Numerous chemical and design strategies have been tested in combine Dicer processing-related potency with increased stability of RNAi drug candidates that include small segmented siRNAs (division into 2 fragments precedes their association with RISC) or incorporating motives that eliminate the Dicer cleavage requirement, but sustain Dicer-RISC interaction [25, 124]. Single-stranded RNAs (ssRNAs) may also be used as RNAi triggers, but their potencies are usually much lower than those reported for dsRNAs [125, 126]. Nevertheless, recent reports show that ssRNAs offer enhanced delivery properties (even entering cells via gymnosis), due to their amphiphilic nature and enhanced structural flexibility (ssRNA vs dsRNA) [125–127]. A similar rule applies to synthetic miRNAs (mimics), and despite ssRNAs containing the sequences that are identical to the guide strands of the mature miRNAs that can function as miRNA mimics, their potency is 100 to 1000 times lower than that of dsRNAs containing miRNAs’ guide and passenger strands [27, 115, 128]. Other strategies include designing longer synthetic miRNA precursors such as pre-miRNA (that will undergo Dicer processing in the cytoplasm) and pri-miRNA (that will require delivery to the nucleus for processing) [129–131].

Another family of RNAi drug candidates, antagomiRs (antimiRs), are synthetic chemically modified ssRNA, about 21-23 nucleotides long, which fully complement miRNAs and effectively sequester mature miRNA in competition with cellular target mRNAs leading to functional inhibition of miRNA [132–137]. However, assessing antagomiRs efficiency in preventing miRNAs activity may be very challenging since their mechanism of miRNA inhibition depends on the type of chemical modifications used. Two types of modified antimiRs can be discussed here: high affinity oligo nucleotides which sequester the targeted miRNA in a heteroduplex, and lower affinity oligonucleotides which promote miRNA degradation as also do cholesterol-conjugated antimiRs [138–141].

Finally, an alternate and more explicit concept relies on the prevention of miRNA interaction with an individual seed sequence of a specific mRNA using target protectors [142]. Target protectors (morpholinos) are chemically modified ssRNAs (~ 25 base) complementary to an mRNA target sequence (at least 14-15 contiguous bases) that prevent the interaction of the miRNA with its specific target, and assembly of the RISC complex [142–145]. The chemical modification of target protectors prevents them from triggering the RNAi pathway, whereas their uncharged backbone facilitates their delivery by non-toxic endocytosis assisted delivery reagents [146].

### Sequence optimization

The potency of the RNAi drug candidate varies greatly depending on its sequence and this ensures specific selection of an antisense strand and minimal off-target effects [147]. Hence, the first concern is the quality of the software package for designing RNAi drug candidates and to predict their efficacy [31, 148–151]. Particularly important in this design procedure is avoiding sequence related off-target effects that may result from partial homology to other transcripts and thus induce miRNA-like activity [152–154]. It has been reported that siRNA duplexes can have differing activities contingent upon the number, position, and base-pair composition of mismatches with respect to the target RNA [155], but so far this problem remains mostly unsolved. Notably, siRNAs seed regions consist of 7 nucleotides, which often results in a large number of partially complementary off-target transcripts. However, modern algorithms often include and develop filtering of siRNA with seed regions that mirror naturally occurring miRNAs and select these with the fewest seed region matches in the 3′ UTRs of off-target transcripts [148, 156].

Since the majority of RNAi drug candidates are dsRNA, both strands can enter RISC. However, on-target silencing requires the guide strand (antisense strand) to remain associated to the active RISC to guide it to the target mRNA, while the passenger strand is degraded and discarded [157]. An improper RISC loading orientation causes the expected guide strand to be neglected and off-target effects are created as the remaining strand is complementary to the unintended transcripts resulting in off-target effects. The same problems apply to synthetic miRNAs, where wrong strand selection at RISCs results in the other miRNA (star form) to be a guide RNAi toward its targets [158, 159]. However, the strand with weaker base pairing at the 5′ terminus of an miRNA or siRNA duplex will be preferred as a guide strand [158, 160]. Furthermore, since a strand with a relatively unstable 5′ end is selected as the guide strand while the strand with a more stable 5′ end is discarded as the passenger strand, the so-called “asymmetry rule” can be applied, by designing a 5′ of the antisense (guide) strand more AU rich than the corresponding end of the sense strand (5′) [157]. Furthermore, AGO proteins display a preference for selecting, as the guide strand, the strand with a U (or less preferably, an A) at position one at the 5′ end. Hence, the ideal passenger strand should consist of C or G at the 5′ end to reduce the risk of selection, whereas the guide strand should contain a U or A at the 5′ end [157]. Furthermore, since there are reports that siRNAs with a rich G/C content are less potent, due to their increased overall duplex thermodynamic stability [147, 161], it is generally accepted as optimal that the G/C content of siRNA is between 30 and 64% [162]. Moreover, sequences with G/C stretches of nine or more nucleotides may reduce the gene silencing efficiency of siRNA and thus should be avoided [163].

Mammalian cells recognize dsRNAs by dsRNA-binding proteins and Toll-like receptors, which results in overall stoppage of protein synthesis and activation of the interferon response [164]. Despite the fact that initial studies assigned activation of the immune response to dsRNAs longer than 30 bp [116], some shorter siRNAs and miRNAs analogs have also been shown to activate innate immunity in a sequence-dependent manner [165, 166]. Notably, the length of the dsRNA threshold may vary among cell types, and even 23 bp siRNAs have been shown to induce interferon responses in some cell lines [167]. To date, several immune-related sequence motifs have been reported to activate Toll-like receptor (TRL) signaling. Unfortunately, these motifs are usually U-rich (e.g. 5′GUCCUUCAA3′, 5′UGUGU3′, 5′UGU3′, or 5′UGGC3′), and thus are hard to eliminate from an RNAi drug candidate sequence [168–171]. Alternate solutions to this problem focus on use of chemical modifications and non-endosomal delivery routes (e.g., electroporation), to prevent TRL activation [172, 173].

Taken together, the development of bioinformatics tools accompanied by better understanding of a drug candidate sequence’s relationship to its potency and specificity has facilitated efficient design of RNAi drug candidates. Nevertheless, such bioinformatic tools depend on the quality of the data deposited in sequence databases (often updated, and problematic in regard to non-coding regions and longer sequence repeats [174]). It should be noted that numerous miRNAs have been recently recognized as sequencing artifacts [175–177]. Furthermore, rules allowing *motifs inducing stress response pathways to be avoided are still poorl*y understood. Therefore, extensive experimental validation of RNAi drug candidate sequence specificity and related off-targets as well as for any possible immunostimulatory adverse effects seems absolutely mandatory. Especially, some off-target siRNA effects can be reduced at concentrations that match the individual potency of these RNAs [178]. However, the recent rapid development and decreasing costs of next generation sequencing, and thus the ability to access entire transcriptome changes upon RNAi drug candidate administration, should facilitate the process of identification and selection of the best candidates with minimal adverse effects.

### Chemical modifications

The vulnerability of RNAs to degradation by endogenous and exogenous nucleases [179, 180], resulting in poor pharmacokinetics, is another obstacle to RNAi therapy. Furthermore, although the right sequence optimization of RNAi drug candidates can greatly improve their specificity and potency and minimize the risk of adverse effects, it cannot completely eliminate the risk of immune response activation [25, 27]. Addressing these issues had resulted in development of numerous chemical modifications that, besides increasing RNAs stability and attenuating immune responses, can also enhance guide strand selection and delivery, as well as reduce RNAi off-target activity [25, 27, 181]. Finally, chemical modifications can be used to facilitate RNAi drug delivery [25, 27, 182]. Importantly, modifications to siRNAs and miRNAs analogs cannot impair their ability to effectively enter and function in the RNAi pathway, and thus prevent their interaction with Dicer and Ago proteins or compromise their silencing efficiency. Since the 5′ phosphate, the 5′ proximal part, and the central positions of the guide strand are crucial for interaction with the RISC, these sites cannot be easily modified [183]. On the other hand, alterations at the whole passenger strand and the 3′ proximal part and 3′ overhang of the guide strand are generally well tolerated [180]. Furthermore, in the case of pri-miRNA chemical modifications should allow nuclear processing [115]. As mentioned above, chemical modifications of antagomiRs determine microRNA fate by targeting it for degradation or accumulation in heteroduplexes [115, 138–141]. Interestingly, possibilities of piRNA chemical modifications are much less well explored, despite the fact that naturally existing piRNAs incorporate the 3′-end 2′-*O*-methyl modification that protects them from RNases [184].

Nowadays, numerous chemical strategies relying on base, sugar or backbone modifications of antisense strands are applied to improve RNAi drug candidate function and stability. Commonly used modifications employ ribose 2′-OH group substitution with other groups along with 2′-O-methyl (2′-O-Me), 2′-fluoro (2′-F) and 2′-methoxyethyl (2′-O-MOE) to increase RNAs nuclease resistance and reduce the risk of immune responses (by preventing TRL activation) [185–188]. However, these modifications may occasionally limit silencing efficiency [189–191].Similar advantages can be achieved by using locked nucleic acid *(*LNA) and unlocked nucleic acid (UNA) modifications that can also minimize the risk of off-target effects by ensuring proper guide strand selection. *L*NA creates a stable “locked” ring conformation by introducing into nucleic acid a methylene bridge between the 2′-O and the 4′-C of pentose [192]. Since LNA modification at the 5′ end of the passenger strand prevents incorporation into the RISC, it reduces the risk of off-target effects [193]. This modification also improves RNA stability and reduces its immunogenicity [165, 193]. However, LNA modifications are also reported to reduce siRNA potency [194].

UNA are based on removal of the C2′ and C3′-bond of the RNA ribose, which decreases modified RNA binding affinity to their target RNAs [195, 196]. Thus, UNA modifications in a seed region of the guide strand can be used to decrease sequence mismatch tolerance, and thus prevent miRNA-like off-target effects [195, 196]. Although single UNA modifications are generally well-tolerated in both the passenger and guide strands and improve RNA, UNA modifications of guide strands can also reduce silencing efficiency [195, 196].

Another chemical strategy relies on substituting the phosphodiester backbone linkages with other types of linkage. In the most common approach, the nonbridging phosphate oxygen atoms are substituted with a sulfur atom to create phosphorothioate (PS) [197]. PS significantly increases the stability of modified RNAs and enhances their pharmacokinetics via promotion of nonspecific binding to plasma proteins [198, 199]. However, in order to maintain RNAi drug candidate compatibility with the RISC pathway, only partial PS modification can be introduced, leaving the center region of the RNA duplex unmodified [200, 201]. Notably, PS modifications along with cholesterol conjugation improve systemic circulation of dsRNAs and stimulate their uptake by gymnosis [124, 202–204]. Furthermore, replacement of siRNA backbone phosphodiesters with the neutral phosphothioesters facilitates such an RNA cellular uptake, while cytosolic thioesterases revert this to native form (short interfering ribonucleic neutrals, siRNNs) [204]. Other chemical strategies are also reported to increase nuclease resistance and accordingly modulate the binding strength with target RNA by using peptide nucleic acids, (PNA) or morpholinos [205, 206].

Taken together, it is clear that optimal pharmacological results and potency of RNAi drug candidates can be obtained by combining the above-mentioned different chemical strategies [207, 208]. Although challenging, as in the classical drug development pipeline, sequential selection and optimization of differentially modified derivatives increases the chance of selecting the leader combination of chemical modifications in terms of stability, potency and specificity.

### Targeted delivery

The cellular membrane constitutes a barrier preventing siRNA and miRNA analogs from entering the cytoplasm, due to their hydrophilic nature, size (~ 14-15 kDa) and negative charge. Moreover, naked nucleic acid molecules are the subject of rapid degradation in biological fluids and, following systemic administration, do not accumulate in target tissue. Hence, the efficient and targeted delivery of RNAi drug candidates still remains one of the major obstacles to the development of RNAi therapies [209]. It is also evident that an optimal delivery system cannot increase toxicity or induce immune response. Furthermore, an optimal carrier should also protect RNAi drugs from degradation in the circulation at physiological conditions and prevent their clearance by the mononuclear phagocytic system, and finally, it should allow their efficient endosomal escape into the cytosol [19, 182, 210].

Many initial clinical approaches and research reports have been based on DNA strategy and viral delivery in which RNAi drug candidates (including miRNA) are produced by intracellular processing of vectors encoding longer RNA hairpin transcripts [211–218]. Following transcription and processing, resultant short hairpin RNAs (shRNAs) and pre-miRNAs enter the RNAi pathway [219, 220]. While research use of this delivery strategy is relatively simple and efficient, and has a large potential for related gene therapy, in a clinical setting usage of viral-derived vectors raises serious concerns regarding their high immunogenicity and the risk of insertional mutagenesis [221–229].

Therefore, chemical excipients have become the leading strategy for delivering RNAi drugs, due to their better safety profile and lower production cost [25, 27, 40, 41, 115, 157, 219]. Furthermore, these chemical carriers/excipients can be modified to accomplish site-specific delivery (by incorporating targeting ligands, as summarized in [230]), or to enhance serum stability [209]. Polymer-based and lipid-based systems are the two main categories of RNA delivery systems.

In lipid-based systems several approaches are used. The simplest are so-called lipoplexes which are cationic lipid-RNA or DNA complexes and which, although successfully used in in vitro studies, appear toxic when administered into animals. More elaborated are long-circulating liposomes (~ 100 nm in diameter) containing either cationic lipid-nucleic acid (lipoplexes) or cationic polymer-nucleic acid complexes inside the liposome water space [231, 232]. RNA-containing lipoplexes or vesicles are taken up by cells mostly via endocytosis and released into the cytosol via the “endosomal escape” pathway [209, 232–234]. However, the lead technology for the non-viral delivery systems of genetic drugs is the so-called lipid nanoparticle system (LNPs) which is based on the method developed by Curtis et al. employing an ethanol-loading procedure, usage of ionizable cationic lipids and rapid mixing [235]. The resulting structure is a ~ 100 nm diameter particle covered with a PEG-lipid monolayer interacting with other constituent lipids in which water-filled cavities containing nucleic acid molecules can be seen [235]. This system has been found to be very efficient in hepatocyte transfection due to liver accumulation and interaction with ApoE. The efficiency of such a construct is high (0.005 mg siRNA/kg body weight in mice); the authors suggest that this is due to “the combination of the optimized cationic lipid MC3, cholesterol and DSPC, together with the rapidly dissociating PEGC14-lipid” [236] The above-mentioned properties and in addition tolerability led to the development the recently approved first RNAi drug, patisiran, directed against transthyretin-induced amyloidosis [23, 24].

In polymer-based delivery systems, cationic polymers are used to establish electrostatic polyplexes with the negatively charged RNA; for example synthetic polyethylenimine (PEI), cyclodextrins, Poly(lactic-co-glycolic acid) (PLGA) and Silica-based nanoparticles) [237–252]. Also, cationic proteins such as protamine and peptides, such as nona-arginine (9R) peptide can be used [253]. An interesting possibility is offered by the application of recombinant protamine as a fusion protein with an scFv antibody fragment which assures targeting of a protamine-nucleic acid complex against cells exposing particular marker molecules to the potential to be bound [254].

All the above-mentioned nanosized particles can enter cells via endocytosis and often promote endosomal escape. However, as mentioned above, because of their high charge density, some cationic nanoparticles are often toxic [27, 255]. Recently, natural cationic polymers such as chitosan, (derived from chitin), and atelocollagen, which is a protein obtained from calf dermis, have been proposed as dependable options for RNA delivery [255–258].

Finally, lipolyplexes consisting of both polymers and lipids are currently being developed to overcome the restraints of the exclusive polymer-based or lipid-based delivery system [249, 259–261].

Importantly, the success of therapeutic RNAi is also often highly dependent on tissue or cell type specific targeting, and thus avoiding unwanted on-target activity in non-target tissues. Target gene expression may be deregulated in target tissue (e.g., cancer cells), but at the same time at the correct levels in healthy non-target tissue (e.g., normal cells). Hence, modulating target gene expression in order to obtain therapeutic benefits in target tissue may be accompanied by deregulation of this gene expression in non-target tissues, leading to toxicity. This is especially important during systemic RNAi drug delivery, since its accumulation in tissues not intended for its activity may be toxic [262]. Furthermore, since miRNA expression is very often tissue and cell type specific [263, 264], targeted delivery is the key to the best potency and minimal off-target effects of related drugs. Hence, the development of targeting ligands for RNAi drugs (e.g., antibodies, aptamers, or small molecules, N-Acetylgalactosamine-GalNAc), as well as methods for their systemic and local administration create another major bottleneck in the further expansion of RNAi therapies [25, 26, 265–270].

## Concluding notes and future prospects

**Fig. 1.**
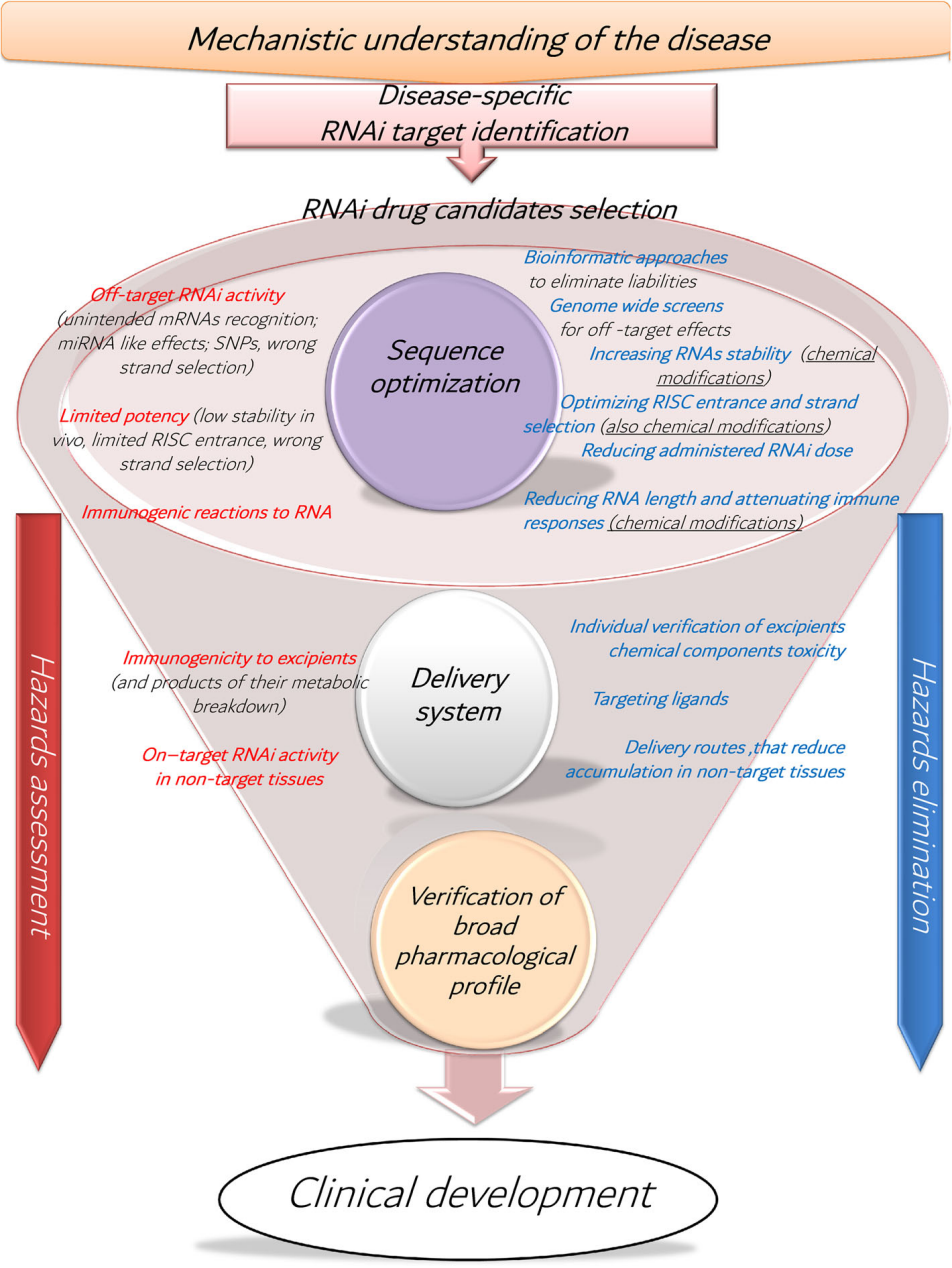
Schematic representation of the RNAi drug discovery and development process. Full mechanistic understanding of the disease allows selection of highly disease specific therapy targets, and thus early elimination of off-targets. In the first phase, candidate sequence design and optimization allows early hazard identification and elimination, whereas chemical modifications can be applied to design out potential hazards and limitations. Furthermore, in later phases potential liabilities regarding delivery system choice should be assessed. Finally, broad pharmacological profiles of the lead drug candidates should be obtained, before drug candidates undergo further clinical development

It is clear that current progress in the RNAi therapy field provides an opportunity to deliver novel drugs that could change patients’ lives. However, despite the success story of Partisiran and multiple other RNAi drug candidates currently progressing through clinical trials, several technical barriers and hazards (Fig. 1) need to be overcome so such therapies could become common clinical treatment; that is, also accessible for orphan diseases.

From the clinical point of view, finding novel effective methods for systemic delivery of RNAi drugs to non-liver and non-kidney tissues, along with dedicated improvement of their pharmacokinetic and pharmacodynamics, remains one of the key challenges in achieving this goal [25, 182, 271]. Hopefully, further development of chemical modifications, as well as better understanding of cellular pathways governing endosomal escape and endocytosis [272–274], will eventually address this issue [275–279]. Finally, although the first RNAi drug is approved, we are very far from understanding the long-term effects of siRNA and miRNA therapy in vivo in human subjects.

Another main challenge is reducing the risks of RNAi drug candidate off-target effects. The initial hazards related to RNAs chemical modifications, non-viral delivery systems and immunogenicity can often be identified, tested in animal models and finally eliminated through the classical drug development pipelines that include in vitro pharmacology profiling [45, 280–283].

Nevertheless, the specific RNA sequences remain the main components and sources of hazards for the drug candidates. Bioinformatics tools try to prevent the design of siRNA with a seed region that is partially complementary with off-target transcripts, but they cannot fully eliminate the risks of all off-target interactions. Although small activating RNAs (ssRNAs) that are structurally identical to siRNA and that can mediate promoter sequence specific activation of some gene expression are also considered in therapy [284, 285], they also represent clear proof of siRNA related hazards. Furthermore, these prediction algorithms are based on consensus genome sequences, and do not eliminate the potential complications related to the occurrence of single nucleotide polymorphisms (SNPs) [67, 68, 286, 287]**.** As estimated for the human genome, SNP can occur once in every 300 bp in both coding and non-coding regions of genes [288], resulting in synonymous and non-synonymous changes that are often reflected in RNA sequences [289]. One such nucleotide change in the human genome may eliminate siRNA or miRNA seed region interaction with target RNA, or result in off-target degradations, as well as disturb miRNA biogenesis [67, 68, 286]. Hence, early detection of SNP-related off-target effects as well as paying attention to population stratification [290–294] are crucial to prevent RNAi drug candidate halt during clinical trials or even its market withdrawal.

For similar reasons to the siRNA/miRNA target sequence specificity, the use of in vivo translational models is very limited and does not allow fair assessment of such a drug candidate toxicity or off-target effects [295]. Furthermore, mRNAs and ncRNAs expression is often sex, age, organ or tissue specific, and thus preclinical development of RNAi drugs requires wide-ranging in vitro studies in different models to prevent both its off-target and on-target activities in non-target tissues [296–300]. Fortunately, recent development and decreasing costs of high-throughput genotyping technologies such as deep sequencing and single cell sequencing [301–304] should allow development of RNAi sequence design and related in vitro pharmacological profiling. Notably, these technologies should propel development of miRNA therapeutics, by advancing understanding of the mechanisms by which these RNAs modulate complex physiological [49, 98, 305–320] and pathological molecular networks [24, 43, 44, 91, 310, 321–359].

Furthermore, long non-coding RNA (lncRNA)-dependent modulation of miRNA levels may become a promising siRNA therapy target [360–370]. However, the biological roles of these ncRNAs, and thus potential off-target effects of lncRNA related therapies, require better understanding [310, 371–384].

Taken together, the critical challenge in the RNAi therapeutics field is the development of highly efficient pipelines for cost-effective selection of RNAi drug candidates that will also allow reduction of safety-related drug attrition. However, overcoming this challenge requires better understanding and more open cooperation between both drug developers and academic researchers. Although basic research studies commonly utilize siRNAs and miRNA analogs to increase our understanding of molecular mechanisms governing human health, they often focus on simplified (single pathway limited) models and thus are difficult to transfer into drug development processes [48, 148, 271, 310, 385–397]. It also has to be stressed that the bioinformatics databases used to predict siRNA/miRNA consequences are generally solely based on scientific literature, and thus are only as valid and efficient as the research underpinning them. However, the scientific literature lacks negative data on ncRNAs function (due to publishing limitations); while the related high scale of comprehensive analysis of publicly deposited genome-wide transcriptomics data is very challenging due to the need for harmonization of transcriptomic approaches and statistical analyses [398–401]. Effectively, the general knowledge obtained by pharmaceutical companies during unsuccessful clinical trials or during general RNAi drug design processes is rarely shared with academics [25, 182, 281–283, 402].

Obviously, closer cooperation between the academic research and pharmacy business realms would help RNAi therapy to realize its full potential to benefit patients.

## Data Availability

Not applicable.

## Abbreviations

Ago2: Argonaute 2
dsRNA: double stranded RNA
EMA: European Medicine Agency
FDA: US Food and Drug Administration
hATTR: hereditary transthyretin amyloidosis
LNA: locked nucleic acid
LNP: lipid nanoparticle system
miRNA: microRNA
ncRNA: non-coding RNA
nt: nucleotide
piRNA: piwi-interacting RNA
PNA: peptide nucleic acids
RNAi: RNA interference
shRNA: short hairpin RNA
siRNA: small interfering RNA
SNP: single nucleotide polymorphism
ssRNA: single-stranded RNA
TRL: Toll-like receptor
TTR: transthyretin
UNA: unlocked nucleic acid
